# Supplementary material for: Multi-trait Polygenic Profiling and Survival Free of Dementia and Disability: Results from the Health and Retirement Study
Source: medRxiv. 2026 Sep 11:2026.09.09.26362686. Preprint. [Version 1] doi: 10.64898/2026.09.09.26362686 (PMC13622800; doi:10.64898/2026.09.09.26362686)
Supplement: Supplementary file 1 [file media-1.pdf]

Supplementary Material

Multi-trait Polygenic Profiling and Survival Free of Dementia and Disability: Results from the Health and Retirement Study

Supplementary Table 1. Interaction between continuous iPRS-DEM and APOE ε4 status by outcome

Outcome	Interaction β (95% CI)	P value
Composite	0.005 (-0.017 to 0.026)	0.674
Dementia	0.025 (0.005 to 0.045)	0.016
Disability	0.014 (-0.005 to 0.032)	0.142
Death	0.009 (-0.013 to 0.031)	0.433

Supplementary Table 2. Time-stratified sensitivity analysis for the composite outcome

Follow-up period	Contrast	HR (95% CI)	P value
0-10 years	Intermediate vs Favorable	1.25 (1.09-1.43)	0.001
0-10 years	Poor vs Favorable	1.67 (1.32-2.12)	<0.001
>10-20 years	Intermediate vs Favorable	1.07 (0.98-1.18)	0.134
>10-20 years	Poor vs Favorable	1.29 (1.05-1.59)	0.016
>20 years	Intermediate vs Favorable	1.15 (1.03-1.28)	0.014
>20 years	Poor vs Favorable	1.43 (1.12-1.82)	0.004

Cox models adjusted for age, sex, genetic ancestry, and PC1-PC10. Favorable polygenic profile was the reference category. HR, hazard ratio; CI, confidence interval; PC, principal component.

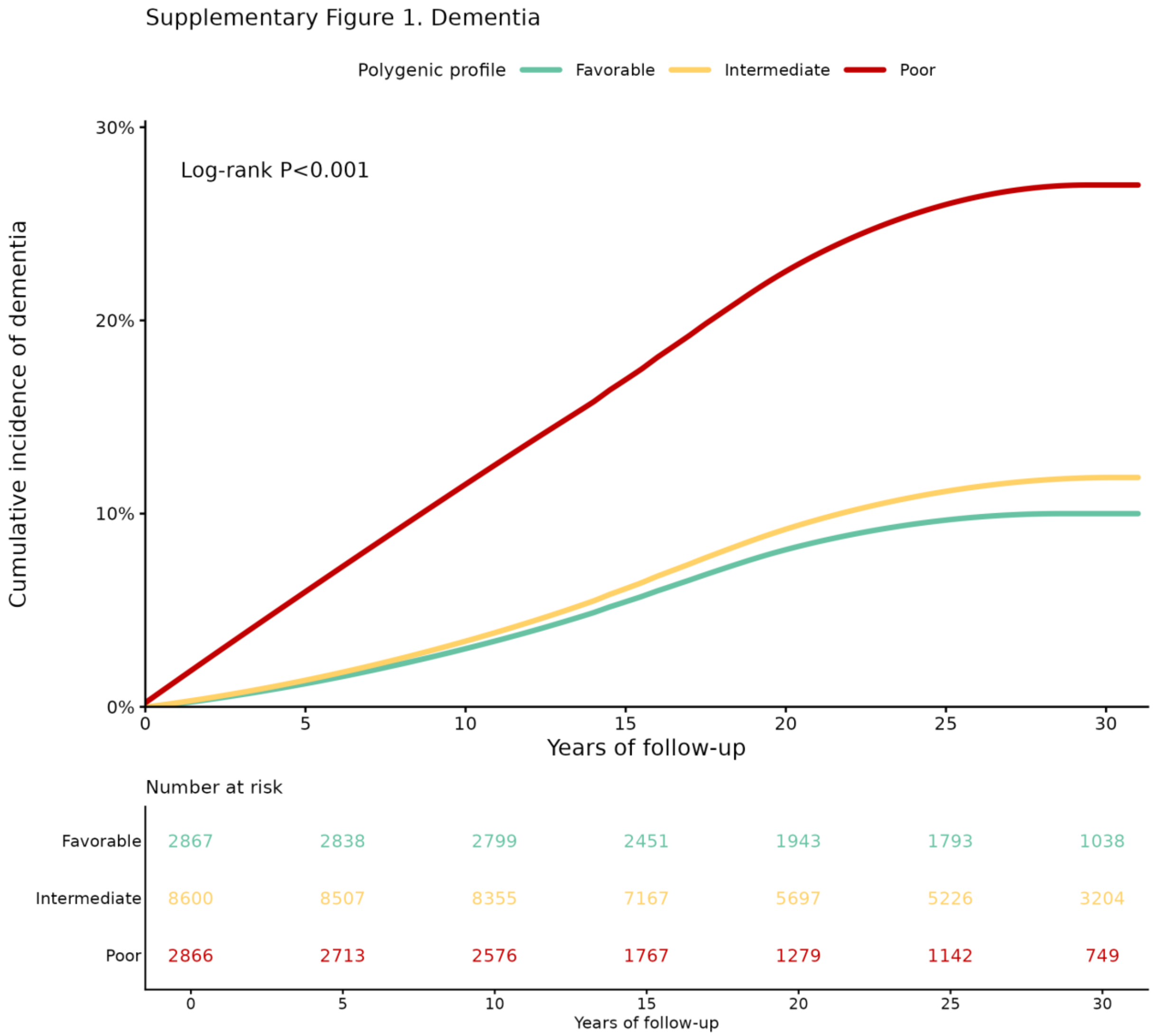

Supplementary Figure 1. Cumulative incidence of dementia according to iPRS-DEM polygenic profile. Numbers at risk are shown below the plot; P value is from the log-rank test.

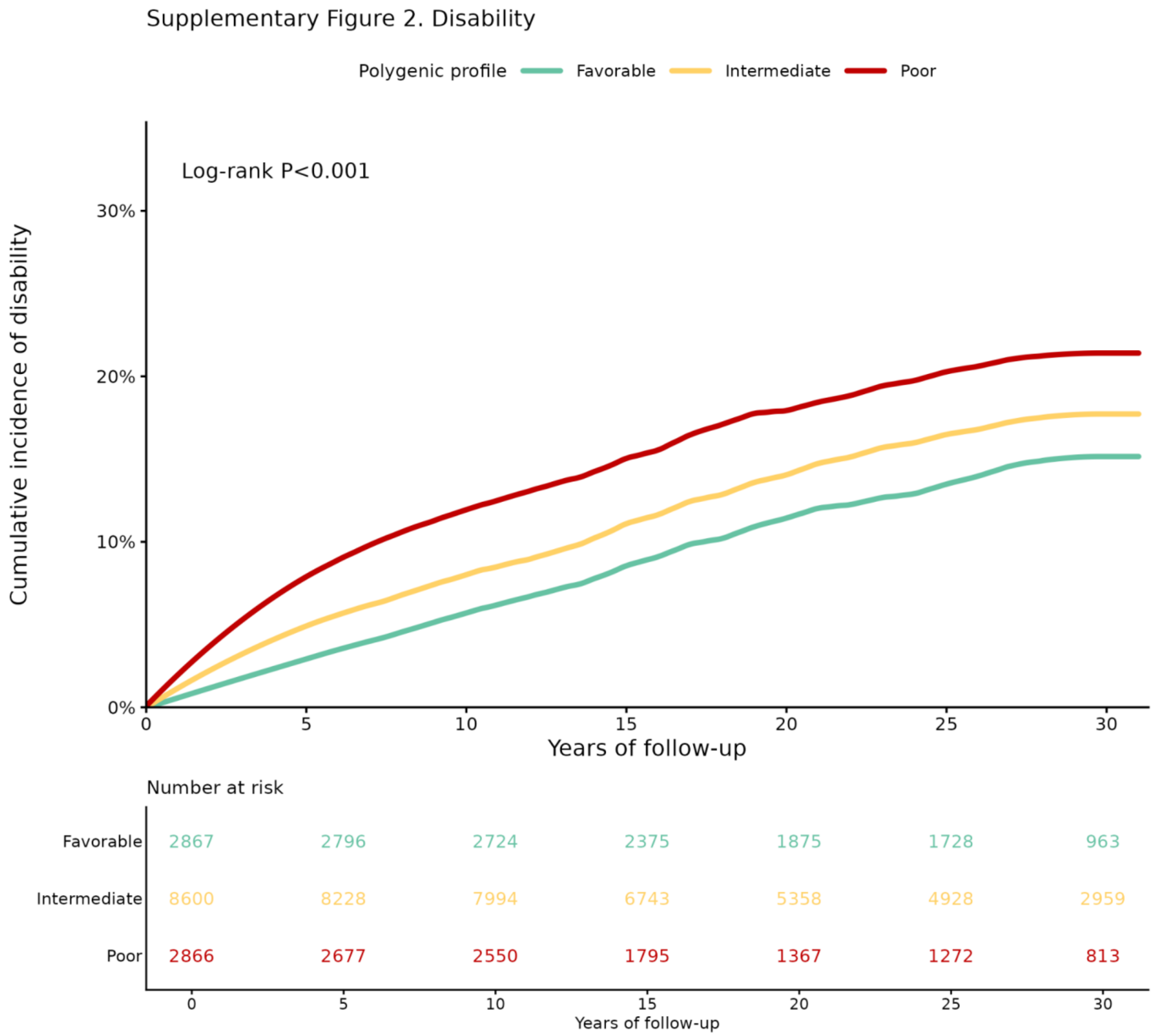

Supplementary Figure 2. Cumulative incidence of disability according to iPRS-DEM polygenic profile. Numbers at risk are shown below the plot; P value is from the log-rank test.

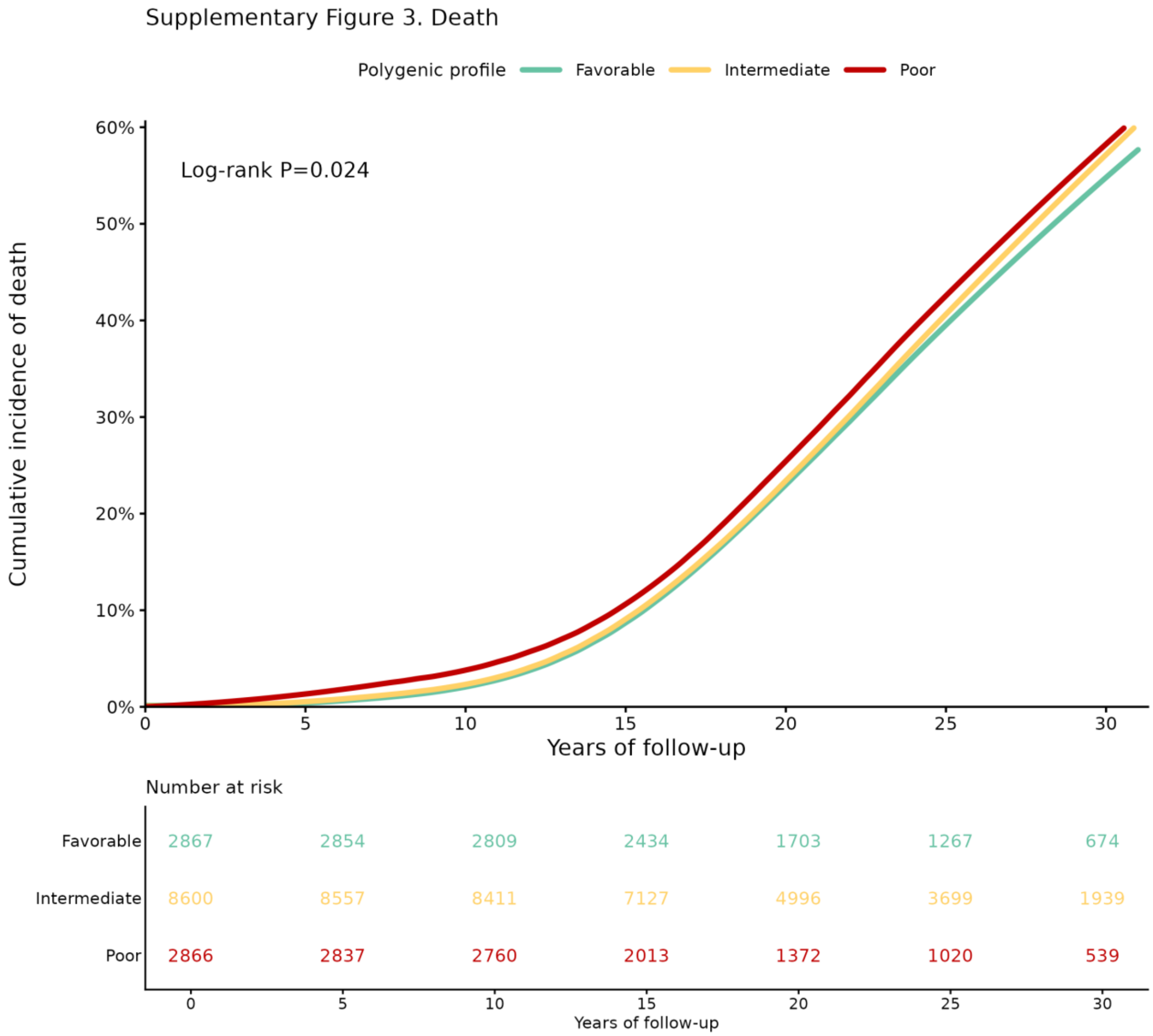

Supplementary Figure 3. Cumulative incidence of death according to iPRS-DEM polygenic profile. Numbers at risk are shown below the plot; P value is from the log-rank test.

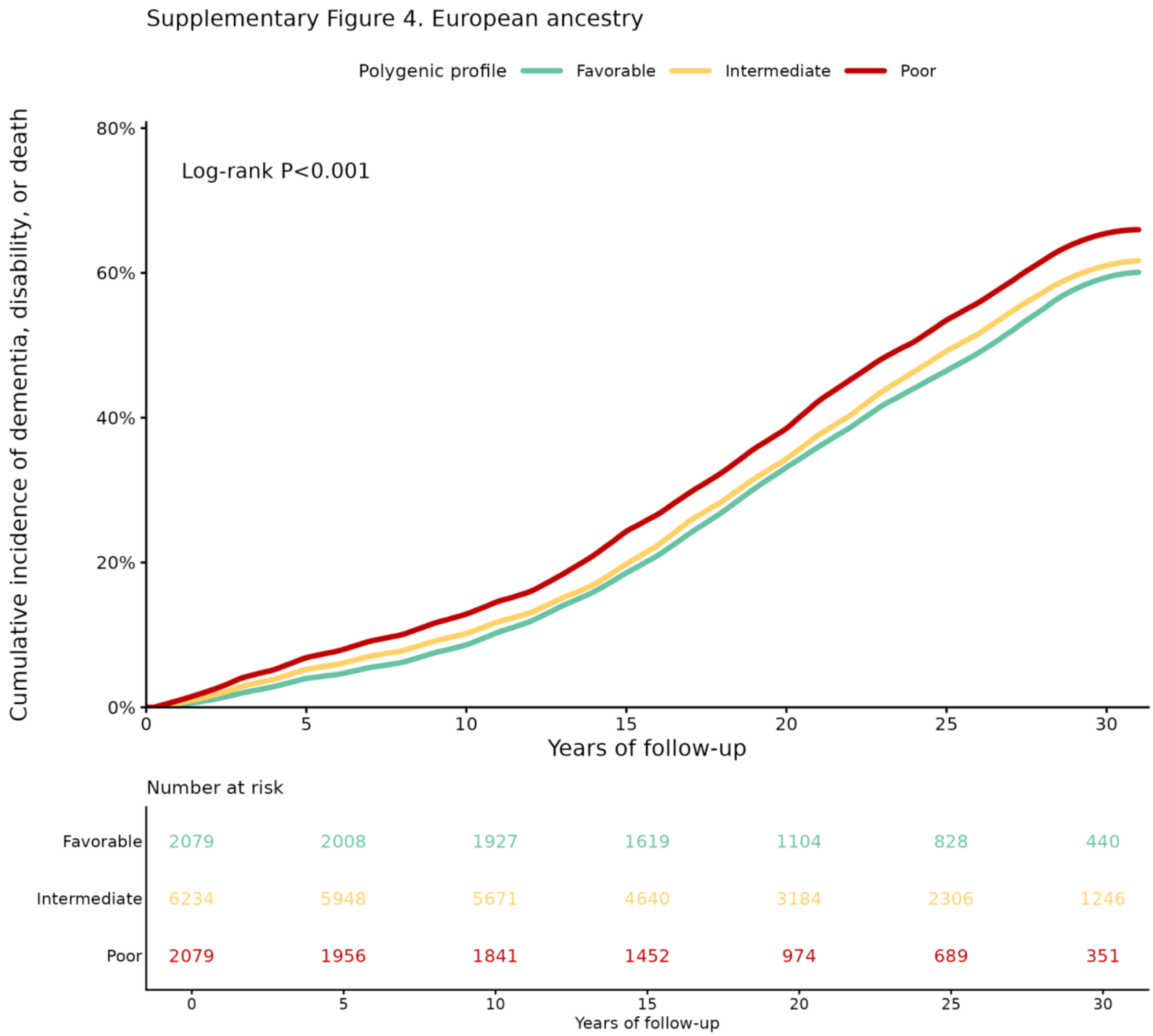

Supplementary Figure 4. Cumulative incidence of dementia, disability, or death according to iPRS-DEM polygenic profile among participants of European ancestry. Polygenic profile categories were defined using within-European-ancestry percentile cut points. Numbers at risk are shown below the plot; P value is from the log-rank test.

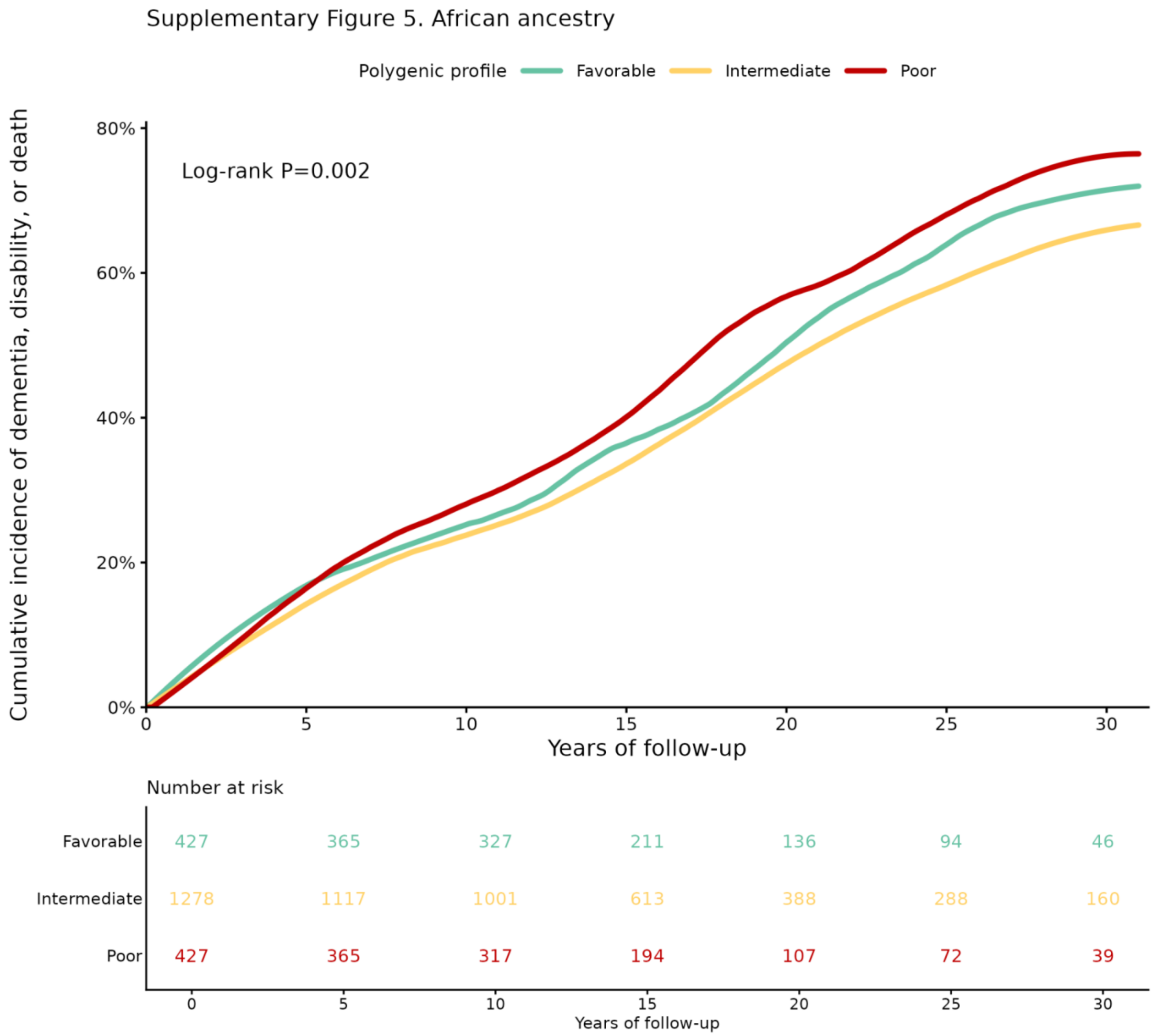

Supplementary Figure 5. Cumulative incidence of dementia, disability, or death according to iPRS-DEM polygenic profile among participants of African ancestry. Polygenic profile categories were defined using within-African-ancestry percentile cut points. Numbers at risk are shown below the plot; P value is from the log-rank test.

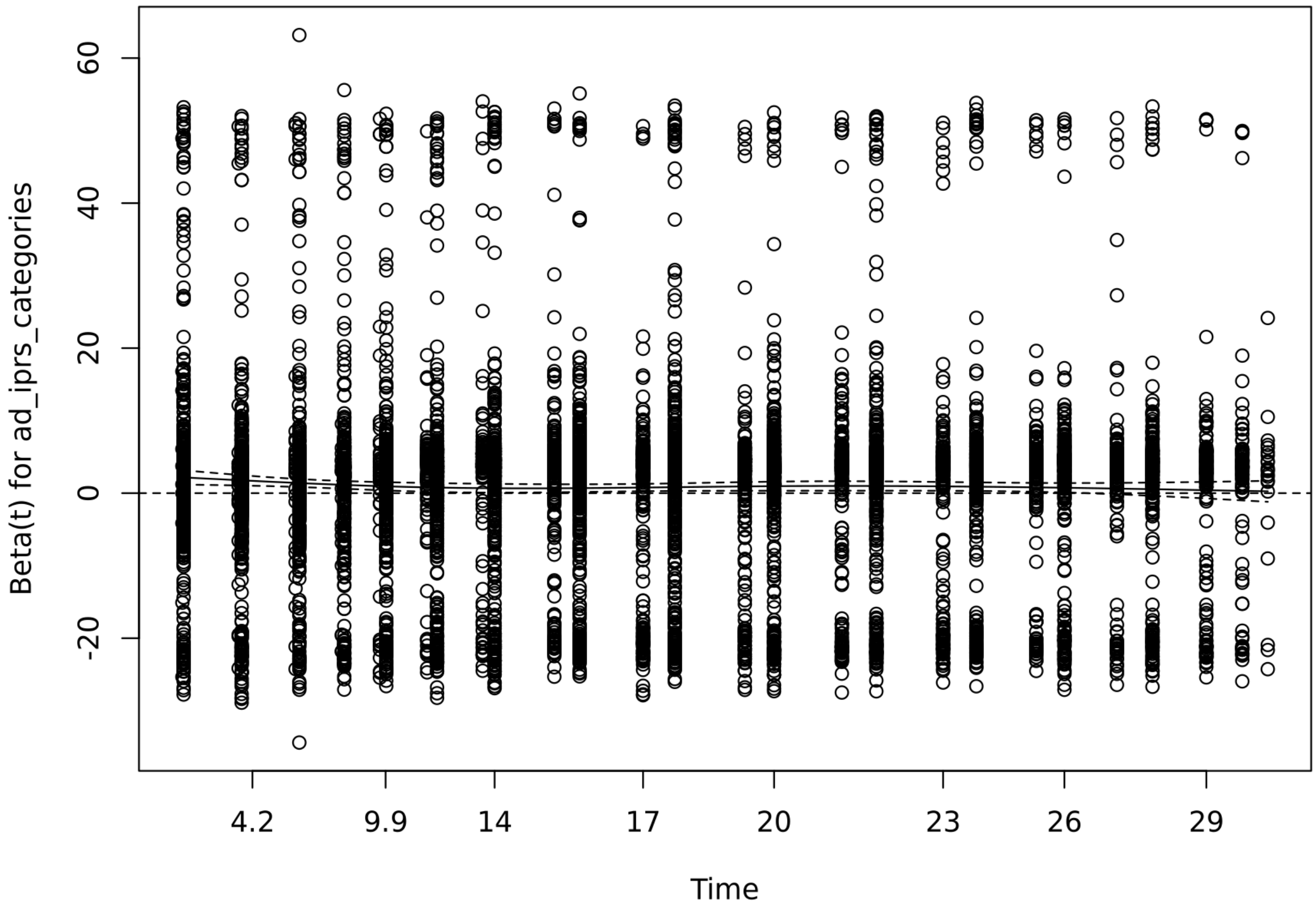

Supplementary Figure 6. Scaled Schoenfeld residual diagnostic for the primary polygenic-profile term in the composite-outcome Cox model. The horizontal reference line at zero and smoothed residual trend are shown to assess time-varying effects.

Abbreviations: APOE, apolipoprotein E; iPRS-DEM, integrated polygenic risk score for dementia.
